# Awareness, use, and need of smart care for older adults: A comparative study based on a survey in Macao, China

**DOI:** 10.3389/fpubh.2023.1135164

**Published:** 2023-04-05

**Authors:** Shiyan Lou, Hong Liu

**Affiliations:** ^1^Faculty of Finance, City University of Macau, Macao, Macao SAR, China; ^2^Bryant University-BITZH Program, Beijing Institute of Technology Zhuhai, Zhuhai, Guangdong, China

**Keywords:** smart care for older adults, smart senior care goods and services, need for smart senior care, awareness of smart senior care, use of smart senior care, caregivers

## Abstract

**Introduction:**

With population aging, taking care of the older adults has become a challenge for many countries and regions, and smart care for older adults is considered as a solution to this problem. Need is an important factor that determines whether the model of smart care for older adults can be efficiently promoted and ultimately help in effective senior care.

**Methods:**

Based on a survey among older adults and their caregivers in Macao, this paper conducted comparative and correlation studies on the awareness, use, and need of smart care goods and services for older adults.

**Results:**

Differences appeared among the groups based on the different categories and items of goods and services. The respondents’ awareness, use, and need of smart senior care were generally low, and levels were lower among older adults than among caregivers; older adults were more concerned about spiritual needs, while the caregivers were more concerned about the health and safety of older adults. Correlation studies showed that awareness of smart senior care was positively related to its use, and these two variables were positively correlated to the need for smart senior care.

**Implications:**

This paper fills in the research gap on the need for smart senior care and can help to promote matching supply and demand to enhance the development of the smart senior care industry.

## 1. Introduction

Population aging is creating a great challenge worldwide, including in the Macao Special Administration Region (SAR), China. In 2021, older adults aged 65 and above accounted for 14.59% of the local population, with an aging ratio of 83.7% and an older adult dependency ratio of 21.5%: Macao is already an aging society. According to the forecast of the Statistics and Census Service of Macao, the local older adult population in Macao will reach 164,400 in 2041, the older adult dependency ratio will be 30.5%, and the aging index could reach 198.8.^1^ Increasingly serious population aging will inevitably bring about increased social security burden, reduced labor force, and unsustainable socioeconomic development, among which how older adults will be cared for has become an economic and social issue that deserves more attention. Especially, the support the older adults can obtain from their children are limited in Macao. For instance, among the 3,608 persons with self-care disability and aged 60 and above, only 25.2% live with their children.^2^

The society and government of Macao SAR have been committed to solving this problem, and the continuous development of information technology has provided new opportunities. Since 1999, with the development of Macao’s economy and the increase of public finance, the local government has paid increasing attention to improving residents’ livelihood and well-being, and senior care is an important part of this. While increasing various forms of economic support, the Macao SAR government has also taken measures to improve services for older adults, encourage their employment, and construct homes for older adults. Since 2017, the Macao SAR government has been committed to a policy of strengthening urban planning and smart city construction; the 2021 Policy proposed smart senior care for the first time, and promised to “develop, as soon as possible, the design and construction of the residence for older adults in Lote-P da Areia Preta, as well as a plan for the services and smart care that goes with it.”^3^

Macao has many advantages in developing smart senior care, including, first, a fairly high income level among residents; in recent years, the median monthly income of the local employed population in Macao has remained at 20,000 patacas (roughly US$2,500). Second, Macao is a small city with a total area of 33 square kilometers, and the income gap is also small with no regional difference, which makes it easy to implement smart senior care. Third, public financing is abundant, which can provide sufficient financial support for smart senior care as a public service. Macao is thus expected to take the lead in realizing smart senior care, if there is sufficient need.

The supply of smart care should be in line with the needs of older adults (1), as this is an important factor in determining whether this model can be efficiently promoted and effectively help older adults. Current awareness and use of smart senior care goods and services (SG&Ss, with singular form SG&S) by older adults and their caregivers is likely to affect their need. Based on the questionnaire survey data, this paper studied the correlation between awareness, use, and need of SG&Ss, which will help to show the current development and need of smart senior care in Macao. The results will be of great significance in optimizing the top-level design of the smart senior care system and improving the promotion efficiency of the model. Because caregivers and older adults are in different age groups and play different roles, their awareness, use, acceptance and need of SG&Ss could vary widely. This paper therefore engaged in a comparative study of these two groups, which contributes to advancing the targeted proposals.

## 2. Literature review

The predecessor of smart senior care was the “completely smart senior care system,” which was first proposed by British Life Trust (2). Then, in 2008, IBM put forward the vision of a smarter planet^4^. Similar plans were advocated by other companies such as Microsoft, Google, Nokia, HP Labs, and NASA (3). Under these circumstances, concepts such as the smart city, smart transportation, smart medical care, and smart senior care came into being. Although there is no conclusive definition of smart senior care, flexibility and cleverness are highlighted (2). This paper defines smart senior care as a model of service provided by modern technology such as the Internet, the Internet of Things, cloud computing, and big data—that is, smart care achieves the purpose of caring for older adults through intelligent means.

One of the biggest problems with traditional senior care is the mismatch between supply and demand, leaving the needs of older adults poorly met. Smart senior care can better match the two sides by collecting a large amount of information about older adults’ needs. However, the collection of massive information is based on the use of SG&Ss, so it is first necessary to determine what factors affect the use of SG&Ss among older adults and their caregivers.

As they age, older adults are faced with more challenges such as mobility and communication, as well as physical, emotional, and mental health (4). Li and Zhang (1) suggested that health, safety, independence, and nursing are essential needs for older adults. Poor social perception and multiple stakeholders have a negative impact on the demand for smart home care (5). It is well known that satisfaction with goods and services affects consumers’ use of them, and Zhang and Li (6) found that health, economic, and family status were important factors affecting satisfaction with smart home-based care among older adults. The development of SG&Ss has been faced with such challenges as intelligent products not being able to solve the just-needed, user-consumption concept and ability to pay (7).

Considering that smart senior care is based on modern technology, the awareness and use of smart senior care by older people and their caregivers are important factors determining their needs. Currently, there is a lack of research on the relationship between these three variables. There is also a lack of research on this topic specifically related to Macao. This paper takes older adults and caregivers in Macao as the research object to investigate their awareness, use, and need of smart senior care as well as the correlation between these three variables.

## 3. Methodology

The data used were acquired through a questionnaire survey. There is no ready-made and suitable questionnaire about the need for smart senior care, so when designing the questionnaires, this paper combined information gathered from the existing literature, the SG&Ss existing in Macao and Mainland China, the ADL assessment scale for health status, on-the-spot investigation on the enterprises and departments supplying SG&Ss, opinions from consultants and experts, initial interviews with older adults and caregivers, and local characteristics of Macao, among other sources. After repeated discussions and revisions, satisfactory questionnaires were finally formed. There were two questionnaires applied in this study: one for caregivers and one for older adults. The main content of the two questionnaires was similar, including the awareness and views of the interviewees related to smart senior care, as well as the health status, living conditions, and personal characteristics of older adults. The caregiver questionnaire also included a few questions about their personal information. This paper focuses on analyzing the core part of the questionnaire, namely the awareness, use, and need of SG&Ss in Macao.

### 3.1. Exploratory reliability and validity test of the questionnaire

An exploratory reliability and validity test of the scales was undertaken with a sample of 50 caregivers and 51 older people respondents. The scale of awareness, use, and needs of caregivers or older adults listed 27 SG&Ss and asked the respondents to answer one by one. The Cronbach’s α value was used as the measurement in the exploratory reliability analysis. The reliability of the scale of the two questionnaires was 0.942 and 0.954, respectively; the reliability is greater than 0.9 (inclusive). Many scholars have pointed out that reliability is acceptable when Cronbach’s α is greater than 0.6 (8–10), so the two questionnaires appear to have good reliability.

Statistical tests were applied to analyze the questionnaires’ validity. The KMO value of the caregiver questionnaire was significantly greater than 0.7, which meant that the questionnaire had good validity; the KMO value of older adults questionnaire was 0.698, which is higher than the value of 0.5 that many scholars find acceptable (11–16). The validity of the questionnaire for older adults was also deemed acceptable.

### 3.2. Questionnaire collection

The research team conducted the survey from November 2021 to January 2022. The research objects included older adults and caregivers of older adults. The convenience sampling method and a combination of face-to-face and online surveys were adopted, because it was difficult to find enough respondents, especially during the COVID-19 period. In the sampling process, the research team tried to ensure the diversity and wide coverage of the sample. The research team contacted almost all senior care institutions in Macao, and six of them responded and provided assistance. The team members also carried out the survey in several parks. To obtain as much information as possible about older adults who were incapable of going out, priority was given to the caregiver questionnaire in the face-to-face survey, regardless of the age of the interviewees. At the same time, most of the caregiver questionnaires were collected through the internet, because most caregivers are busy with work and tend to use the internet and mobile phones. In the face-to-face surveys, the team members read the questions and options in Cantonese for the respondents and marked their choices.

A total of 306 samples were collected, 11 unqualified samples were eliminated, and 295 valid samples were obtained. Finally, there were 204 older adults samples (200 face-to-face interviews and 4 online samples) and 91 caregiver samples (18 face-to-face interviews and 73 online samples). During the survey, almost all of the respondents were very cooperative, but the list of questions was long and some respondents had never considered a few of the questions; some high quality samples therefore contained one or two missing items, which were regarded as qualified ones when conducting statistical analysis. The confirmatory reliability analysis showed that the combined reliability of the scale was 0.970 and 0.976 for the samples of older people respondents and their caregivers, respectively, and the reliability reached the standard.

The core part of this survey was the awareness, use, and need of SG&Ss, containing 27 items divided into five categories. The first category was daily-life care (10 items) on topics such as online shopping, intelligent daily facilities, and public service platforms. The second category was healthcare (9 items) on, for example, telemedicine and smart medical wearable facilities. The third category was safety (3 items) on real-time activity recognition, positioning, and remote monitoring. The fourth category was spirituality and emotion (3 items) on online entertainment, online communication and social interaction, and chatbots and electronic photo albums. The fifth category was self-actualization (2 items) on online learning and online acquisition of social activity information.

## 4. Survey results: Analysis

For the 204 valid samples of older adults, males accounted for 26% and females for 73% of respondents; this is consistent with the fact that the average life expectancy of women is longer than men’s. Younger older adults occupied the majority in terms of age, with 30% aged 65–69, 24% aged 70–74, 29% aged 75–84, and 17% aged 85 years old or above. Nearly 70% had primary school education or below, about 20% had junior high school education, and about 10% had high school education or above; overall, the education level of older adults was relatively low. Their health status was basically good, and 51% of older people respondents believed that their health was “fairly good” or “very good,” while only 12% considered their health to be poor.

Among the 91 valid caregiver samples, 37% were male and 63% were female; the proportion of females was higher than that of males, which could be related to the fact that caregivers are generally female. Most caregivers were middle-aged, with 28% of the caregiver respondents being under 30 years old, 22% aged 30–39 years, 32% aged 40–49 years, and 18% aged 50 years or above. About half of the caregivers had a college degree, followed by those with a high school education, who accounted for about 20%, and those with a master’s degree, accounting for 10%. The education level of the caregivers was thus much higher than that of older adults. More than 60% of caregivers had a full-time job, while full-time students and retirees each accounted for about 10%, and only a small percentage of caregivers were working part-time, unemployed, or had withdrawn from the labor market. Most caregivers therefore care for older adults while working and are thus faced with additional pressure. A rating scale was designed to investigate the awareness, use, and need of senior care goods and services, and it was divided into two parts focused on use and need.

### 4.1. Comparative analysis of awareness and use of SG&Ss

The assessment of SG&S use was sub-divided into two parts. First, the respondents were asked whether they had used the good or service. Then, two options were provided for non-users, which were “I know the smart senior care good or service (SG&S) but have never used it” and “I do not know the SG&S.” If the former was chosen, it meant that the respondent was reluctant to accept SG&S, while if the latter was chosen, it meant that lack of knowledge about the good or service prevented respondents from using them.

#### 4.1.1. Overall awareness and use of SG&Ss

**Figure 1:**
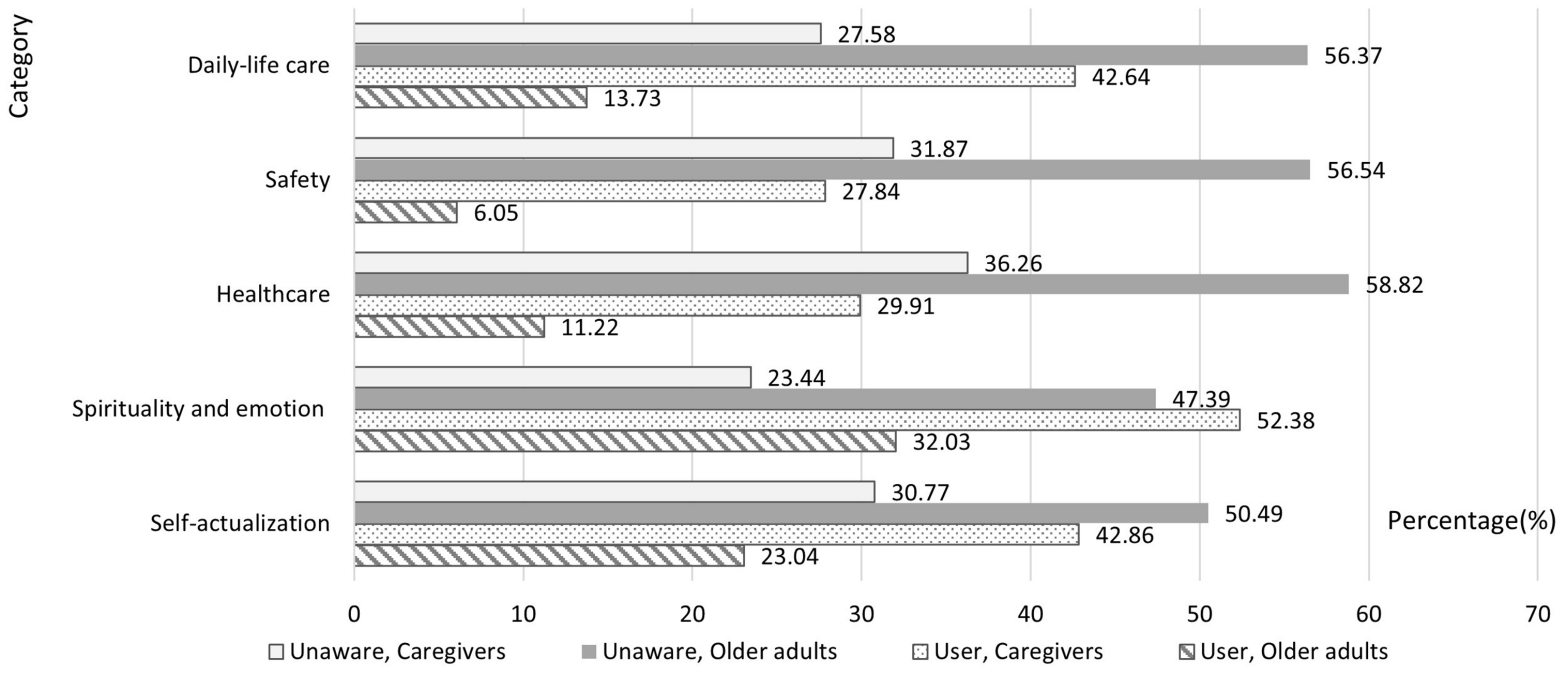
Comparison of the awareness and use of different categories of SG&Ss between older adults and caregivers. A small number of respondents insisted they wanted to “think about it later” or said, “I really do not know, it is difficult to make a judgment,” so there was an average of about one respondent who did not make a choice for each item. The total is the average for the 27 items, with all samples counted in the denominator.

As can be seen in Figure 1, first, from the total average of 27 items, more than 80% of the older adults had never used the SG&Ss listed in the rating scale. About 60% of the caregivers had never used the mentioned SG&Ss. The overall penetration rate of SG&Ss thus appears to be low. At the same time, the proportion of caregivers who had used SG&Ss was 20 percentage points higher than that of older adults. Second, most of the older adults who had not used the SG&Ss did not know about them, accounting for 55.77% of the older adult sample and 66.12% of the non-user older adult samples. Among the caregivers who had not used the SG&Ss, the proportion of those who knew and did not know about them was roughly the same: those who did not know accounted for 30.73% of the entire caregiver sample and 47.78% of the non-user caregiver sample. Most of older adults had poor information channels and did not know about advanced SG&Ss, which was an important factor hindering their use of smart senior care. This was also clearly reflected in the questionnaire survey process. There were some cases in which the older adults expressed that they did not know or understand a certain SG&S, no matter how the investigators explained it and illustrated it with examples. Caregivers could obtain information about SG&Ss through more channels, and the penetration rate of SG&Ss among caregivers was relatively high. However, lack of knowledge remained an important obstacle for caregivers in the use of SG&Ss.

#### 4.1.2. Classification and comparison of awareness and use

SG&Ss are divided into five categories: daily-life care, healthcare, safety, spirituality and emotion, and self-actualization. There are both similarities and differences in the awareness and use among older adults and caregivers in these five categories. First, for both the older adults and their caregivers, the proportions of those who had used SG&Ss in the spirituality and emotion category were the highest, accounting for 32.03 and 52.38%, respectively; the proportions in the safety category were the lowest, 6.05 and 27.84% of older adults and caregivers, respectively. For both older adults and caregivers, the usage rates of the three subitems in the spirituality and emotion category were quite different; the rate of use for online communication and social interaction was very high, with 56.37 and 72.53%, respectively, while the rates of use of chatbot/electronic photo album were as low as 3.43 and 21.98%, respectively. The use ratios of the three security subitems were all low, with little difference between the two groups.

Second, among the older adults and caregivers who had not used SG&Ss, the proportion of those who said “I do not know about it” was the highest for healthcare, accounting for 58.82% of older adults and 36.26% of caregivers, and the lowest for the spirituality and emotion category, accounting for 47.39 and 23.44% of older adults and caregivers, respectively. In general, both groups lacked awareness of SG&Ss in the healthcare category, and the awareness of some subitems was particularly low, and no subitem received a high score. For example, the proportions of those who answered “I do not know” for the listed nine SG&Ss in the healthcare category were all higher than 45 and 20% for older adults and caregivers, respectively, especially for smart beds and telemedicine, with 78.43 and 62.75% of older adults and 47.25 and 42.86% of the caregivers, respectively.

Similar to use, both the older adults and their caregivers showed great internal differences in the awareness of the three subitems in the spirituality and emotion category. Among them, the awareness of online communication and social interaction was high, and the proportions of those who did not know this item were as low as 31.37 and 7.69%, respectively. The corresponding proportions about chatbots/electronic photo albums reached 73.53 and 43.96%, respectively. For both the older adults and caregivers, there was no significant difference in the awareness of the three subitems in the safety category, all of which were poor, perhaps because they believed that medical care is not for “daily use,” so most people have little contact with it in daily life and only people with poor health and their caregivers pay attention. WeChat has been vigorously promoted and popularized in Macao as a communication channel in recent years; chatbots and electronic photo albums are cutting-edge SG&Ss. Obviously, overall awareness and use are positively correlated.

#### 4.1.3. Comparison between awareness and use of different items

Comparing the 27 items, it was found that, first, in terms of awareness, older adults and caregivers had great similarities; for instance, more respondents knew about online communication and social interaction, online shopping, online traffic information and services, and how to search for online information, but far fewer of them knew about smart beds, online rental services for assistive devices, and intelligent shifters. The biggest difference in awareness between the two groups of people appeared for public service platforms: only 18.68% of caregivers were not aware of it, while 61.27% of older adults did not; this difference was far larger than for other SG&Ss. Other items with notable differences included legal aid services and electronic payments/internet banking. The difference between the two groups was the smallest for awareness of first aid.

Second, in terms of the use of SG&Ss, the older adults and caregivers were also fairly consistent. Information inquiry, communication, social interaction, and online entertainment were widely used by both groups, while the use of legal aid services, intelligent shifters, and smart beds was low for both groups. The biggest difference in the usage ratio between the two groups mainly appeared in online shopping, electronic payment/online banking, and public service platforms; the proportions between the two groups differed by 53.70, 49.01 and 37.55 percentage points, respectively.

Although the usage rate of SG&Ss among older adults and caregivers was low in general, for a few items it was high, and for a few others it had developed rapidly. These benefited from both the popularization of intelligent electronic products and vigorous promotion by the Macao SAR government in recent years. In 2017, the Macao SAR launched the building of a smart city. Since then, under the premise of continuous improvement of the relevant systems, enterprises set discounts or offered bonuses to actively promote these systems, and finally realized both the popularization of domestic and international third-party payment *via* platforms such as WeChat Pay, Alipay, and PayPal, as well as the launch and gradual promotion of local online banking, electronic payments, online shopping, and so on. Since the outbreak of COVID-19 in 2020, the government actively promoted internet and mobile smart services, including the Macao Health Code, nucleic acid test booking, vaccination booking, and an anti-epidemic electronic consumption voucher scheme. These greatly promoted the use of smart services in medical care and electronic payments among Macao residents.

It can be seen that both older adults and caregivers generally have a higher ratio of awareness and use of items that are helpful in daily life, supported by convenient apps, and actively promoted by enterprises or the government. The ratios are lower for items that are not commonly used, are cutting-edge or substantially innovative, larger in size, higher in price, or in short supply, such as smart beds and intelligent shifters. For both older adults and caregivers, there is a positive correlation between awareness and use of SG&Ss.

There are, however, big differences between the older adults and the caregivers in the awareness and use of SG&Ss. This may be for several reasons. First, caregivers and older adults have different abilities to recognize, bear, and avoid risks; for example, banking and online shopping are relatively risky, and the difference between the two groups is large. Second, the division of labor between older adults and caregivers in the family differs. In addition to caring for older adults, caregivers generally have to work, look after other family members, and interact with the outside world. The other household responsibilities older adults undertake in addition to taking care of themselves generally include doing housework and taking care of grandchildren. Third, caregivers tend to be busy, while older adults often have plenty of time; the former thus often use online services to save time, while the latter are usually willing to line up on the spot.

### 4.2. Comparative analysis of the need for SG&Ss

**Figure 2:**
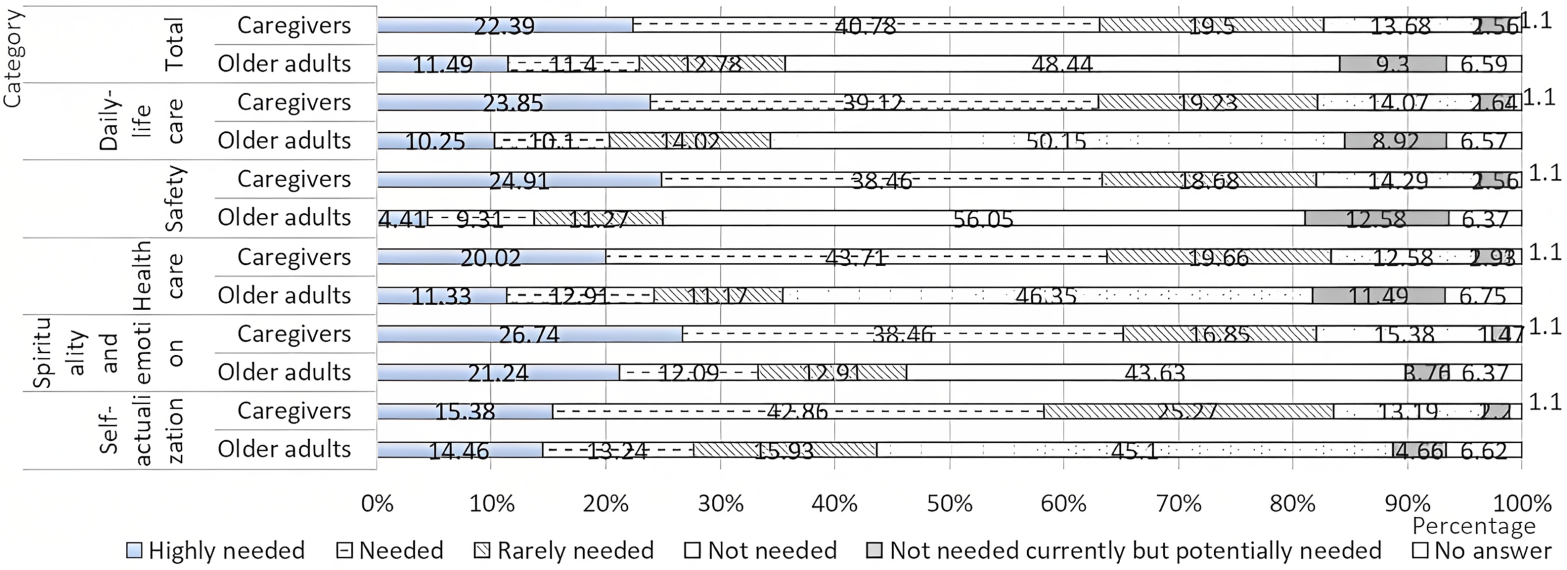
Comparison of the needs of older adults and caregivers for different categories of SG&Ss.

Regarding the respondents’ need for each SG&S at present and in the future, five options were provided: highly needed both currently and in the future, needed both currently and in the future, rarely needed both currently and in the future, not needed either currently or in the future, and not needed currently but potentially needed. The statistical results show that the levels of demand among older adults and caregivers are very different in general and for different categories, as shown in Figure 2.

First, the overall average of the 27 items shows that caregivers had a much higher need for SG&Ss than older adults. The listed SG&Ss were “highly needed” or “needed” by only 22.89% of the older adults, while the corresponding percentage of caregivers was 63.17%; and the proportions of “not need” was 48.44 and 13.68%, respectively, with the former being nearly 30 percentage points higher than the latter.

Second, the SG&Ss were divided into five categories. The need of older adults for the spirituality and emotion category was the highest, and the need for safety category was the lowest. The sum of “highly needed” and “needed” accounted for 33.33 and 13.73%, respectively, which revealed that the difference in the need among older adults for different categories of goods and services was large. The need among caregivers for the spirituality and emotion category was also the highest, but the need for self-actualization was the lowest. The sum of “highly needed” and “needed” accounted for 63.74 and 58.24% of responses, respectively, which revealed that the difference in need among caregivers for different categories was small. The differences in need between older adults and caregivers for safety and daily care categories were large, with the percentages of “highly needed” and “needed” of being 49.64 and 42.62 percentage points lower, respectively, for older adults than among caregivers. The differences in need between older adults and caregivers for self-actualization and the spirituality and emotion categories were comparatively small, with the percentages of “highly needed” and “needed” being 30.54 and 31.87 percentage points lower, respectively, for older adults than for caregivers.

The results reflect that older adults pay more attention to their own self-actualization and spiritual and emotional needs, and believe that they can take care of themselves, so they do not want to be “monitored.” Additionally, although caregivers agree that SG&Ss are needed to meet older adults’ spiritual and emotional needs, caregivers generally pay more attention to the daily needs of older adults while neglecting the spiritual pursuit of “self-actualization.”

Third, for each item, both older adults and caregivers had a high need for online communication and social interaction and searching online information, while the need for the other items differed widely, especially electronic payment/online banking, real-time activity recognition, and positioning; the proportion of older adults who needed these three items was 52.67, 51.84, and 51.23 percentage points lower, respectively, than that of caregivers. In terms of SG&Ss that are least needed, the older adults and caregivers had a high consistency—that is, the three least needed items were online legal aid services, chatbots/electronic photo albums, and intelligent shifters.

This is consistent with the conclusion drawn from the analysis above—that is, there is a high need for SG&Ss that facilitate life and have convenient apps; moreover, due to the usually poor health status of older adults, the need for medical care–related items was relatively high, which confirms the necessity of combining medical and senior care. The needs for the items related to high professionalism, low probability of use, and cutting-edge products and services were low. The needs of older adults for more complex, difficult to master, error-prone, and large loss–causing items were much lower than those of caregivers.

### 4.3. Correlation analysis of awareness, use, and need of SG&Ss

A Pearson correlation analysis between awareness, use, and need of SG&Ss was conducted with Stata 17 software. To compare caregivers and older adults, the correlation analysis was done separately. The percentage data were used. The proportion of users for each item was used as a measure of use rate; the proportion of users plus the proportion of “I know the SG&S but have not used it” was the measure of awareness; and need was measured by the proportion of each choice in the questionnaire. The results are shown in Table 1.

**Table 1:** Correlation between awareness, use and need of SG&Ss, older adults and caregivers.

Variables	Older adults		Caregivers	
	Awareness	Use rate	Awareness	Use rate
Use rate	0.100014(0.502589)		0.415436^**^(2.283560)	
Highly needed	0.076006(0.381133)	0.918200^***^(11.58997)	0.603844^***^(3.787741)	0.673542^***^(4.556194)
Needed	0.190311(0.969270)	0.494740^***^(2.846471)	−0.244125(−1.258709)	0.037213(0.186193)
Rarely needed	0.322468(1.703330)	−0.072363(−0.362767)	−0.494118^***^(−2.841737)	−0.473072^***^(−2.684785)
Not needed	−0.228171(−1.171766)	−0.837477^***^(−7.662563)	−0.310884(−1.635461)	−0.749370^***^(−5.658593)
Not needed currently but needed potentially	−0.192071(−0.978573)	−0.627198^***^(−4.026386)	−0.171645(−0.871153)	−0.040379(−0.202058)

The values of the T statistics are in brackets. ^***^ and ^**^ mean significant at the 1% and 5% levels respectively.

For both older adults and caregivers, the use rate had a strong positive correlation with awareness (Pearson correlation = 0.7685 and 0.9295 respectively, greater than 0.5^5^ and significant at 1%). That is, for both older adults and caregivers, increasing their knowledge of SG&Ss would significantly increase the probability of their use. In terms of need of SG&Ss, for older adults, both awareness and use rate exhibited a strong positive correlation with “highly needed” and “needed” (Pearson correlation = 0.7442, 0.9341, 0.5538 and 0.6556 respectively, greater than 0.5 and significant at 1%) and correlated strongly negatively with “not needed” and “not needed currently but needed potentially” (Pearson correlation = −0.7512, −0.8433, −0.6410 and − 0.7199 respectively, with an absolute value greater than 0.5 and significant at 1%). While for caregivers, both awareness and use rate were strongly positively correlated with “highly needed” (Pearson correlation = 0.7384 and 0.6379 respectively, greater than 0.5 and significant at 1%), insignificantly positively correlated with “needed,” moderately negatively correlated with “rarely needed” (Pearson correlation = −0.4936, with an absolute value between 0.3 and 0.5 and significant at 1%; and − 0.4278, with an absolute value between 0.3 and 0.5 and significant at 5%), and strongly negatively correlated with “not needed” and “not needed currently but needed potentially” (Pearson correlation = −0.6516, −0.6781, −0.5052 and − 0.5173 respectively, with an absolute value greater than 0.5 and significant at 1%). Which means that the higher the awareness or the use rate, the higher the percentage of respondents who needed the SG&Ss and the lower the percentage of the respondents who did not need the SG&Ss.

It can be seen that, much similarity exists between older adults and the caregivers as for the correlation between awareness, use and need of SG&Ss. Whether for older adults or the caregivers, there is a positive correlation between these three variables. Getting knowledge of SG&Ss is the first step in giving rise to an idea of the need and use of them. The use of SG&Ss and the mining of their functions can increase the perceived need of older adults and caregivers for them. In short, for both older adults and caregivers, increasing their awareness of SG&Ss can increase not only their use probability but also their need for them, and the use of SG&Ss can also increase their need.

## 5. Discussions

The analysis results showed that most of the respondents had never used the listed SG&Ss, and most of older adults who had never used these items did not know about their existence. Ahn and Aghvami (18) drew a similar conclusion that both caregivers and older adults lack knowledge in smart care service. Meanwhile, caregivers had much higher awareness and use rates for most SG&Ss than older adults. The reason may lie in the age-related digital divide or so-called grey divide (19, 20) and it is widely believed that older adults are characterised by technophobia, digital illiteracy, and non-use of technology (21).

Looking at needs, first, the needs of both the older adults and their caregivers were low, with the needs of older adults tending to be around 40 percentage points lower than for caregivers. This conclusion is partly supported by the study of Sitar-Taut et al. (22) on older adults. The reason may lie in the fact that the technology readiness and technological interactivity of older adults affect the perceived ease of use and perceived usefulness of the SG&Ss and technological anxiety affects users’ intention to use (23). Magdalena et al. (24) proved the importance of familiar, usable, desirable, and cost-effective technologies in increasing the need for smart senior care.

Regarding the five subcategories, the use rate of the spirituality and emotion category was the highest for both older adults and caregivers, while the safety category the lowest. Among non-users, awareness of the healthcare category was the lowest, and the spirituality and emotion category was again the highest. Meanwhile, the need of the older adults for spirituality and emotion was the highest and the need for safety was the lowest. Among caregivers, the need for spirituality and emotion was also the highest and the need for self-actualization category was the lowest. Safety is the concern of caregivers and they also would like to support their older family members to enjoy their life (18).

As for each item, older adults and caregivers had both similarities and differences. In terms of awareness, the main similarity between the two groups was that both groups were most knowledgeable about online communication and social interaction, shopping on online, and searching online information, while they were least knowledgeable about smart beds, online rental services for assistive devices, and intelligent shifters. There was also a great difference between the two groups in awareness of public service platforms, online legal aid services, and electronic payments/online banking. As far as use rate was concerned, there were some broad similarities. Use rates were relatively high in online communication and social interaction, searching network information, online traffic information and services, and online entertainment, while use rates for intelligent shifters, online legal aid services, and smart beds were low. The use rate of each item was higher among caregivers than among older adults, and the difference was the largest in online shopping, electronic payments/online banking, and public service. And the need for each item was higher among caregivers than among older adults. The similarities were that the needs for chatbots/electronic photo albums, intelligent shifters, and online legal aid services were the lowest for each group, while online medical care booking and online communication and social interaction were among the items showing the highest need. In terms of electronic payments/online banking, online shopping, and online activity management, the needs of caregivers were much higher than those of older adults.

In general, those SG&Ss that are widely used in daily life, supported by convenient apps, and related to a low risk threshold are more popular among and of higher need to both older adults and caregivers. Items seldom seen in daily life that are complex, high-risk, high-price and cutting-edge were of low use rate and need, especially for older adults. These findings support Zhou et al. (25), who reported that Trust perception, Ease-of-use perception and Cost perception are among the eight attributes influencing the acceptance of smart senior care. And they are supported by the studies of Eastman and Iyer (26) on the Internet use of the older adults and of Costa et al. (27) who believed that smart TVs were a most convenient solution for the provision of social and health services for older adults since they are most familiar with TV set.

For the older adults and the caregivers, a significant positive relationship was found between awareness, use, and need of SG&Ss. As Jo et al. (28) reported, awareness about the benefits and development of smart senior care can increase the older adults’ adoption of smart service. Zhou et al. (25), Cao et al. (29) and Huang et al. (30) also found that older adults’ experiences in using smart products are positively related to the need of them.

## 6. Conclusion and implications

The need for SG&Ss is an important determinant of adaption and development of the smart senior care model. This paper studied two important factors affecting the need of smart senior care, namely, the awareness and use of SG&Ss. At the same time, the differences between older adults and caregivers in age, education, personal ability, and purpose resulted in great differences in their awareness, use, and need of SG&Ss. This article conducted a questionnaire survey of 27 SG&Ss, the needs of which were divided into five categories that were studied as a whole and separately.

The analysis results showed that, the awareness, use rate and need of both older adults and caregivers were generally low, and those of older adults were much lower than those of caregivers. Regarding the five subcategories, firstly, both older adults and caregivers have a better knowledge of the spirituality and emotion category and a worse knowledge of the healthcare category. Secondly, the use rate of the spirituality and emotion category was the highest for both older adults and caregivers, while the safety category the lowest. Thirdly, the need of both older adults and caregivers for spirituality and emotion was the highest, while the major difference lie in the fact that the need of older adults for safety was the lowest and the need of the caregivers for self-actualization category was the lowest. Awareness, use, and need of SG&Ss are significantly positively correlated for both older adults and caregivers.

Based on the above analysis and conclusions, the following proposals to the government and related enterprises are put forward. First, the security of the platforms and apps related to SG&Ss should be improved by the application of blockchain or other advanced technologies. Second, challenges faced by older adults such as the digital gap and health problems should be taken into consideration, and older-adult-friendly and easy-to-operate goods, platforms, and apps should be designed. Third, publicity should be strengthened. Publicity can increase the use and need of both older adults and the caregivers for SG&Ss. The government can promote the widespread of smart senior care awareness, products, and services by encouraging the Macao media to cooperate with experts, scholars, and enterprises in the field of smart senior care at home and abroad. Fourth, trials should be encouraged and supported. The use of SG&Ss can significantly increase residents’ need for such products. The government can thus encourage enterprises to donate related products and services to senior care institutions for trial use by the older adults and caregivers to create demand by increasing supply. Fifth, the development of leasing businesses should be encouraged. In view of the fact that large-scale smart senior care products, such as smart beds and intelligent shifters, are expensive, bulky, and difficult to deal with when the family no longer needs them, the government can encourage senior care institutions and enterprises to develop related leasing business to reduce the cost to and relieve the worries of families. Sixth, given that caregivers have more knowledge and a higher probability of using SG&Ss while being faster in learning how to use them and having a stronger ability to prevent risks, training caregivers would be beneficial not only in their care-taking of older adults, but also for their future self-care. Therefore, it may be more efficient to train caregivers than older adults.

The major limitation of this article lies in the survey. Although we tried to make the sample more representative, yet those with self-care disability tend to be stay indoors and inadequately represented. And the sample is limited due to the constraint of time and manpower and affected by COVID-19. Further studies are needed with expanded scope of the survey and a more representative sample.

## Data availability statement

The original contributions presented in the study are included in the article/supplementary material, further inquiries can be directed to the corresponding author.

## Author contributions

SL and HL designed the questionnaires. SL led the questionnaire collection, wrote the initial draft of the manuscript, designed the study, and conducted analysis. HL did proofreading. Both authors contributed to writing the final manuscript.

## Funding

This research was supported by the Education Fund of the Macao Special Administrative Region, project number: HSS-CITYU-2020-01.

## Acknowledgments

We thank the following social service centers as well as their leaders and staff offering help during the survey, which include União Geral das Associações dos Moradores de Macau, Centro de Convívio da Associação de Mútuo Auxílio dos Moradores do Sam Pá Mun, Centro para ldosos lat Seng da Taipa da União Geral das Associações dos Moradores de Macau, Lar de ldosos “Hou Kong Yuet Lai”, Complexo de Serviços de Apoio ao Cidadão Sénior “Pou Tai”, Caritas de Macau and so on. We appreciate the hard work of the students who acted as the investigators. And we thank LetPub (www.letpub.com) for its linguistic assistance during the preparation of this manuscript. We thank all the project members for their constructive suggestions, including professors Jianmin Li, Adrian (Wai Kong) Cheung, Weimin Chen, Jinying Wang, and Yang Chen, and Jian Sheng.

## Conflict of interest

The authors declare that the research was conducted in the absence of any commercial or financial relationships that could be construed as a potential conflict of interest.

## Publisher’s note

All claims expressed in this article are solely those of the authors and do not necessarily represent those of their affiliated organizations, or those of the publisher, the editors and the reviewers. Any product that may be evaluated in this article, or claim that may be made by its manufacturer, is not guaranteed or endorsed by the publisher.

## Footnotes

^1^Government of Macao SAR, Statistics and Census Service. Macao Population Projection, 2022–2041. https://www.dsec.gov.mo/zh-MO/Statistic?id=102. February 24, 2023.

^2^Government of Macao SAR, Statistics and Census Service. 2021 Population Census. https://www.dsec.gov.mo/zh-MO/Statistic?id=103. February 24, 2023.

^3^Government of Macao SAR of the People’s Republic of China. Report on the Lines of Government Action for the 2021 Financial Year. November 16, 2020. https://www.gce.gov.mo/content.aspx?l=pt#policy2021_pt. December 18, 2022.

^4^IBM. IBM builds a smarter planet. https://www.ibm.com/smarterplanet/us/en/. December 30, 2022.

^5^The strength of the correlation is strong if the absolute value of Pearson correlation coefficient is greater than 0.5, moderate if between 0.3 and 0.5, weak if between 0 and 0.3 and none if 0. The direction of the correlation is shown by the sign of the coefficient. Source: Turney (17). Retrieved on February 23, 2023.

## Abbreviations

SAR: Special Administration Region
SG&Ss (with singular form SG&S): smart senior care goods and services
